# Medical Application of Polymer-Based Composites

**DOI:** 10.3390/polym12112560

**Published:** 2020-10-31

**Authors:** Haw-Ming Huang

**Affiliations:** 1 School of Dentistry, College of Oral Medicine, Taipei Medical University, Taipei 11031, Taiwan; hhm@tmu.edu.tw; Tel.: +886-291-937-9783; 2 Graduate Institute of Biomedical Optomechatronics, College of Biomedical Engineering, Taipei 11031, Taiwan

Composites are materials composed of two or more different components. When polymers are combined with other materials, properties of individual polymers such as mechanical strength, surface characteristics, and biocompatibility can be improved, making it feasible to manufacture materials with excellent mechanical properties and biological activity. Polymer-based composites are commonly used in aerospace, automobile, military, and sports applications, and are increasingly being used in biomedicine for tissue engineering, wound dressings, drug release, regenerative medicine, dental resin composites, and surgical operations [1,2,3]. Research topics in this area cover technical issues of how best to integrate these materials and postintegration material properties.

Developing biological materials using bioderived polymers as a matrix is another medical application of polymer-based composites. Many recent studies have reported improvement of specific material properties by mixing biocompatible nanoparticles into bioabsorbable polymers [4,5,6]. These nanoparticle/polymer composite biomaterials are showing great promise for use in biomedical applications including tissue engineering [7,8] and wound healing [9,10], and can also be used to fabricate contrast agents for medical imaging in cancer detection or as targeting materials for precise cancer treatment [11]. With this background in mind, this Special Issue entitled Medical Application of Polymer-Based Composites brings together 11 original articles representing recent progress and new developments in the field of biopolymer-based composites.

For tissue engineering researchers working on bone regeneration technology for periodontics, implantology, and maxillofacial surgery, developing new substitute bone grafts that show strong bone growth activity is an ongoing challenge. In this regard, Salamanca et al. developed and tested the performance of collagenated porcine grafts and found that collagenated porcine graft not only induced osteoblast differentiation in vitro, but also exhibited guided bone regeneration in vivo [12]. They demonstrated that collagenated porcine graft has potential to act as an osteoconductive material that can be used for different dental guided bone regeneration procedures.

Poly-L-lactide (PLLA) is a biodegradable polymer widely used in orthopedic devices and dental rehabilitation [13]. Although it exhibits excellent biocompatibility, its insufficient mechanical strength limited its application in load-bearing area. In order to increase the osteoregeneration and accelerate the formation of new bone, bioactive materials have been added to PLLA [14]. For example, to prevent leakage of polymethyl-methacrylate (PMMA) bone cement during kyphoplasty treatment, Leu et al. developed a novel membrane by mixing PLLA and tricalcium silicate as a barrier [15]. They showed that the mechanical and antidegradation properties of this hybrid composite were improved without affecting its cytocompatibility and created a potential antileakage membrane solution for kyphoplasty treatment of osteoporosis related spine fractures.

Soft tissue repair is another important tissue engineering challenge. To develop a collagenase carrier for tendon–bone healing, Huang et al. developed thermosensitive chitosan–gelatin–glycerol phosphate hydrogels. After testing in an animal model, they suggested this novel composite as a potential biomaterial to assist tendon-to-bone healing [16].

Biological polymers are produced naturally in living organisms. They are large molecules composed of many small organic monomers. The use of biological polymers from waste to produce new materials for medical use is a trend for sustainability. Since chitosan and its derivatives have been reported to be a material with high chelating capacity [17], Huang et al. designed an irrigant system using chitosans derived from waste Ganoderma tsugae for smear layer removal in endodontic treatment and concluded that chitosan derived from fungal biomass shows potential as an alternate irrigant for clinical use [18]. Thacker et al.’s study testing physical properties and cell response of artificial tears produced using Bletilla striata polysaccharide is another example of natural plant-based polymers with tissue engineering applications. Their findings indicated that fabricated artificial tears can effectively reduce inflammatory cytokines and reactive oxygen species (ROS) levels in tested cells [19].

Hyaluronic acid (HA) is also a biopolymer found in the various living organisms [20,21,22] that has shown potential for bone regeneration treatment. The primary physiological characteristics of HA is excellent viscoelasticity and water absorption [23]. Chang and his coworkers developed a gelatin/hyaluronate copolymer composite and mixed it with calcium sulfate, hydroxyapatite, and stromal-cell-derived factor-1 for bone regeneration enhancement [24]. After in-vitro mesenchymal stem cell (MSC) tests and an in-vivo rat model experiment, they found that their Gel-HA/CS/HAP/SDF-1 composite showed an obvious regenerative effect for treating bone defects.

Low-molecular-weight HA (LMWHA) has been reported to exhibit anti-inflammatory effects and accelerated wound repair response [25,26,27,28]. To test this, Huang’s group prepared LMWHA by gamma-irradiation [29]. After testing LMWHA’s chemical and physical properties, the material was combined with carboxymethyl cellulose to form a composite. Their animal experiment showed that wounds in animals covered with the CMC/LMWHA composite decreased in size to almost half that of wounds in animals covered with CMC fabric. Their conclusion suggested that this CMC/LMWHA composite can be an excellent wound dressing material for skin injury.

Nanoparticles have seen recent use in biological applications. Nanomagnetite (Fe_3_O_4_) is one of these nanoparticles and has attracted attention due to its biocompatibility and functionality [11,14]. In addition, several investigators have manufactured PLLA-Fe_3_O_4_ composites using electrospinning or injection molding techniques. Since Fe_3_O_4_ nanoparticles exhibit superparamagnetism, the material has been reported to produce a contrast agent for enhancing the quality of MR tumor images [30]. In addition, HA can target tumor cells through CD44 receptors on tumor cell surfaces [31,32]. Accordingly, in 2020, Wang et al. mixed the natural polymer LMWHA with Fe_3_O_4_ nanoparticles to target MCF7 breast cancer cells. Using time-of-flight secondary ion mass spectrometry, they proved that LMWHA-Fe_3_O_4_ nanoparticles have the potential to be used as an injectable agent that can target breast cancer tumors [11].

Drug delivery systems are another application of biopolymer composites. In 2019, Murgia et al. developed a nanostructure lipid carrier to release curcumin [33]. Since curcumin has been reported as a natural polyphenolic compound exhibiting antibacterial, antioxidant, anti-inflammatory, anticancer, and wound healing effects [33,34,35], this delivery system can be used in the treatment of oral diseases such as oral lesions and periodontitis. 

Optically sensitive polymers can also be used as a component to fabricate composites for medical use. Fan et al. developed a new composite using a liquid-crystal-based polydimethylsiloxane substrate. A series of in-vitro tests proved that this design can be used as a detector for multimicrofluidic immunoassays [36]. Bessonov et al. also developed a novel fibroin methacrylamide which can be photocrosslinked into hydrogels and improve their mechanical properties [37]. In addition, their studies showed that their modified polymer increases osteoinductive activity, including increasing cell numbers, rearranging actin cytoskeleton, and improving distribution in focal contacts, indicating that this modified substrate can be used in tissue engineering applications.
